# Chronic intermittent hypoxia accelerates cardiac dysfunction and cardiac remodeling during cardiac pressure overload in mice and can be alleviated by PHD3 overexpression

**DOI:** 10.3389/fcvm.2022.974345

**Published:** 2022-09-12

**Authors:** Xuan Xu, Peng-Hao Zhen, Fu-Chao Yu, Tao Wang, Sheng-Nan Li, Qin Wei, Jia-Yi Tong

**Affiliations:** ^1^Department of Cardiology, Zhongda Hospital, Southeast University, Nanjing, China; ^2^Medical School of Southeast University, Nanjing, China

**Keywords:** chronic intermittent hypoxia, cardiac dysfunction, cardiac remodeling, SERCA2a, HIF-1α

## Abstract

Obstructive sleep apnea (OSA) accelerates the progression of chronic heart failure (CHF). OSA is characterized by chronic intermittent hypoxia (CIH), and CIH exposure accelerates cardiac systolic dysfunction and cardiac remodeling in a cardiac afterload stress mouse model. Mechanistic experiments showed that long-term CIH exposure activated hypoxia-inducible factor 1α (HIF-1α) expression in the mouse heart and upregulated miR-29c expression and that both HIF-1α and miR-29c simultaneously inhibited sarco-/endoplasmic reticulum calcium ATPase 2a (SERCA2a) expression in the mouse heart. Cardiac HIF-1α activation promoted cardiomyocyte hypertrophy. SERCA2a expression was suppressed in mouse heart in middle- and late-stage cardiac afterload stress, and CIH exposure further downregulated SERCA2a expression and accelerated cardiac systolic dysfunction. Prolyl hydroxylases (PHDs) are physiological inhibitors of HIF-1α, and PHD3 is most highly expressed in the heart. Overexpression of PHD3 inhibited CIH-induced HIF-1α activation in the mouse heart while decreasing miR-29c expression, stabilizing the level of SERCA2a. Although PHD3 overexpression did not reduce mortality in mice, it alleviated cardiac systolic dysfunction and cardiac remodeling induced by CIH exposure.

## Introduction

Obstructive sleep apnea (OSA) is a sleep disorder resulting from disordered breathing, and its hallmark feature is chronic intermittent hypoxia (CIH) during sleep (1). OSA is an independent risk factor for serious cardiovascular events and is involved in the occurrence and development of events such as hypertension, myocardial infarction, congestive heart failure and stroke (2). In addition, OSA is associated with adverse cardiac outcomes of chronic heart failure (CHF). According to reports, 20% of symptomatic CHF patients have OSA (3, 4). Notably, large prospective cohort studies have demonstrated an increased incidence of HF in patients with severe OSA (5). Much evidence suggests that the pathophysiological effects of OSA in heart failure (HF) are mediated by multiple mechanisms, including neurohormonal activation, oxidative stress, and inflammation. These effects can accelerate the progression of HF and of symptoms associated with it (6). In animal models, CIH exposure for 5 weeks was shown to increase the heart/body weight ratio, increase the left ventricle (LV)/total heart weight ratio and decrease left ventricular function in SD rats (7). C57BL/6 mice had increased left ventricular perivascular fibrosis after 6 weeks of CIH exposure (8).

Continuous positive airway pressure (CPAP) therapy is currently the main treatment used for sleep apnea. In 2017, the American Heart Association/American College of Cardiology gave CPAP strategies a class IIb recommendation for people with HF and OSA (9). However, meta-analyses have shown that CPAP therapy does not significantly improve the left ventricular ejection fraction (LVEF), number of hospitalizations for HF events, or all-cause mortality in patients with or without HF (10). Furthermore, CPAP therapy appears to be only partially effective in patients with HF and does not reduce the incidence of cardiovascular events in patients with OSA and HF (11). In addition to the limited treatment effect, the poor long-term compliance of patients with CPAP therapy, which is only 40%-80%, is a limitation of this treatment modality (12). CPAP therapy is indeed an effective treatment for OSA, but effective combination therapy therapies are still needed to improve the adverse cardiac consequences of OSA.

Hypoxia-inducible factor 1 (HIF-1) is a transcription factor that consists of two subunits: HIF-1α, whose expression is regulated by O^2^, and HIF-1β, which is constitutively expressed (13). Clinical studies have shown that the expression of HIF-1 is upregulated in the organs, tissues, and circulating blood of OSA patients (14, 15). In an animal model of cardiac pressure overload, the long-term elevation of cardiac-specific HIF-1α expression was found to accelerate the impairment of cardiac function (16). In a cardiomyocyte hypertrophy model, HIF-1α transcriptional activation was shown to promote the cardiomyocyte hypertrophy induced by Ang II (17, 18). Ang II or transverse aortic constriction (TAC) surgery-induced cardiac pressure overload in animals can lead to HF. Oxygen-derived radicals, especially superoxide anion, produced during CIH lead to HIF-1α upregulation and its long-term high expression in cardiac tissue (19, 20). We therefore speculate that CIH exposure could accelerate cardiac pressure overload-induced HF and that HIF-1α may be a new therapeutic target.

Our previous study showed that HIF-1α upregulation during intermittent hypoxia induces endothelial cell mesenchymalization and thus increases cardiac fibrosis and cardiac dysfunction (21). To alleviate the effects of HIF-1α, we overexpressed PHD3, which is a physiological inhibitor of HIF-1α that is abundantly expressed in the heart (22). We found that inhibition of HIF-1α by overexpression of PHD3 reduced endothelial mesenchymalization and improved fibrosis and systolic function. However, the study was limited to endothelial cells and did not involve cardiomyocytes. Notably, PHD3 was also overexpressed in cardiomyocytes in the study, and cardiomyocytes had a greater effect on myocardial contractile function. Therefore, we wanted to further explore whether the overexpression of PHD3 in cardiomyocytes can mitigate the adverse cardiac effects of CIH.

In the present study, we investigated the mechanisms underlying the CIH acceleration of cardiac injury in TAC mice and the involvement of HIF-1α in this pathology. We explored the molecular basis associated with HIF-1α function and the possible therapeutic role of PHD3.

## Methods

### Animals

**Figure 1:**
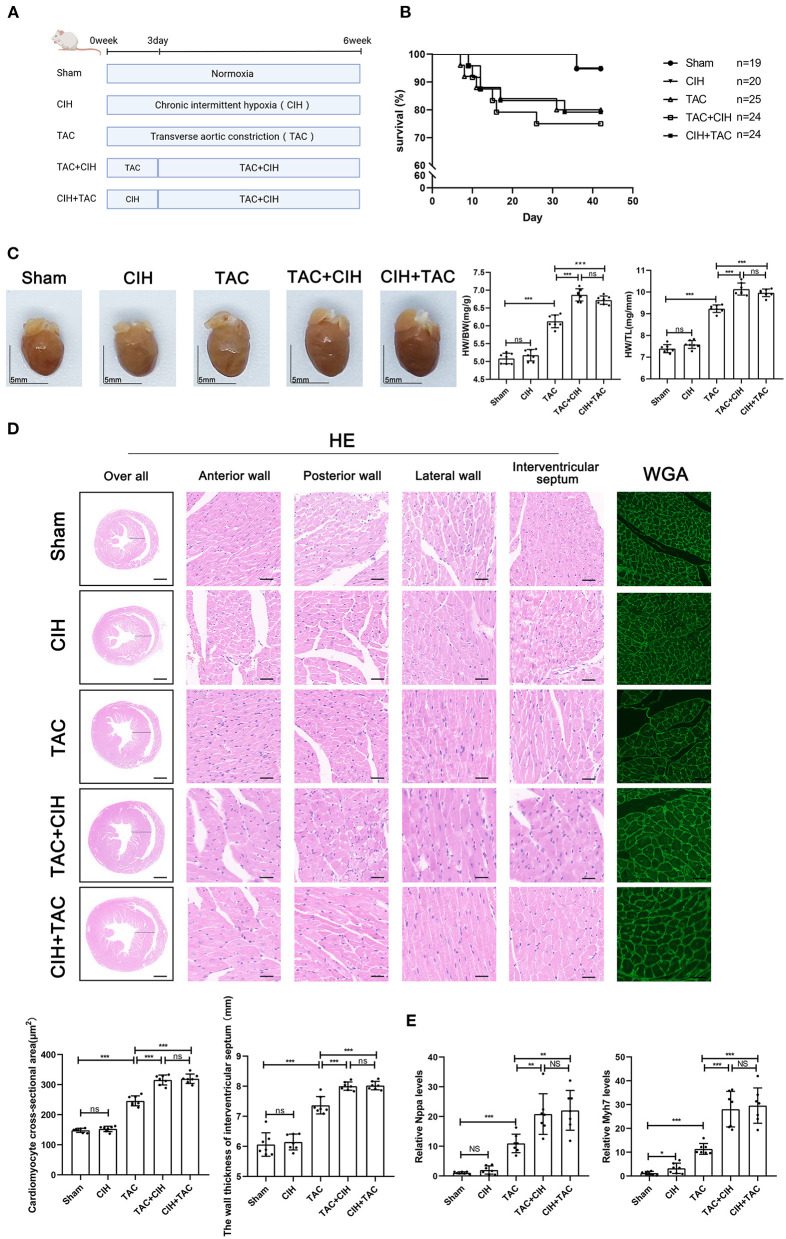
CIH exposure does not affect basal mortality rates after TAC but promotes cardiomyocyte hypertrophy. **(A)** Grouping and experimental protocols for each group. Sham: thoracotomy only without transverse aortic constriction surgery, followed by 6 weeks in normoxia; CIH: thoracotomy only without transverse aortic constriction surgery, followed by 6 weeks in CIH exposure; TAC: transverse aortic constriction surgery, followed by normoxia for 6 weeks; TAC+CIH: CIH exposure was performed 3 days after transverse aortic constriction surgery for a total of 6 weeks. CIH+TAC: transverse aortic constriction surgery was performed 3 days after CIH exposure and CIH exposure was continued for 6 weeks thereafter. **(B)** Survival rates of all groups; the sample size of each group is noted in the figure. **(C)** Left: representative images of hearts; right: quantification of the heart weight-to-body weight ratio and heart weight-to-tibia length ratio. *n* = 7; scale bar = 5 mm; ***p < 0.001. **(D)** HE staining and WGA staining of mouse hearts. The wall thickness of interventricular septum was measured according to HE staining pictures. The cardiomyocyte cross-sectional area was quantified according to WGA staining. *n* = 7; scale bar = 1 mm and 25 μm; ***p < 0.001. **(E)** Real-time PCR analysis of relative Nppa and Myh7 levels in heart tissue at week 6. *n* = 7; *p < 0.05, **p < 0.01, ***p < 0.001.

A total of 351 10-week-old male C57BL/6 mice were purchased from the Animal Experimental Middle School of Southeast University. For details on the experimental procedures and groups, please refer to Supplementary Figures 1, 2. All animal experiments were carried out in accordance with the guidelines for the care and use of laboratory animals formulated by the Animal Care and Use Committee of Southeast University School of Medicine. The TAC and CIH exposure protocols were approved by the Animal Experiment Ethics Committee of Southeast University School of Medicine. The afterload stress mouse model was established as reported (23). In short, mice were anesthetized with isoflurane (3% isoflurane for induction, 2% isoflurane for maintenance). The aorta was ligated with a bent 26-G needle and a 6–0 silk suture between the origin of the right innominate and left common carotid arteries, and then the needle was removed to induce narrowing of the blood vessel. Buprenorphine (0.01 mg/kg) was given subcutaneously for postoperative analgesia. The mouse was kept warm until it recovered from the anesthesia. CIH exposure was performed as described (21). Mice were placed in a plexiglass container (48^*^33^*^23 cm, 5 mice in each container) with a constant temperature of 22–24°C. Nitrogen was introduced to reduce the oxygen concentration in the container to 4–5% within 8 s, and it was kept there for 12 s. Then air was introduced to restore the oxygen concentration to 21% within 40 s. This made for a 60-s cycle. The mice were exposed to this intermittent hypoxia treatment for 8 h/day during the 12-h dark period. CIH animals were kept in a normoxic environment during the 12-h light period. When investigating the mechanism, we divided the mice into five groups (Figure 1A): (1) Sham group: Only thoracotomy. The mice were placed in containers with free air circulation for 8 h/day for 6 weeks according to the protocol. (2) CIH group: Only thoracotomy. The protocol for CIH exposure was followed for 6 weeks. (3) TAC group: After TAC surgery, the mice were placed in containers with free air circulation for 8 h/day for 6 weeks according to the protocol. (4) TAC+CIH group: Three days after TAC surgery, CIH exposure was performed according to the protocol (a total of 6 weeks from the time of TAC surgery). (5) CIH+TAC group: The mice were first exposed to CIH for 3 days and then underwent TAC surgery. They were submitted to CIH again after surgery (a total of 6 weeks from the time of first CIH exposure). When exploring the effect of PHD3 treatment, we also divided the mice into five groups (**Figure 5A**): (1) TAC group: The protocol was the same as above. (2) CIH control group: CIH exposure was performed 3 days after TAC surgery, and CIH exposure was stopped after 2 weeks (a total of 6 weeks from the time of TAC surgery). The goal was to simulate cases in which OSA is cured during CHF. (3) TAC+CIH group: The protocol was the same as above. (4) Adeno-associated virus 9 (AAV9)-NC group: CIH exposure was performed 3 days after TAC surgery, followed by tail vein injection of AAV9-vehicle (5 × 10^11^ vg) 1 week later (a total of 6 weeks from the time of TAC surgery). (5) AAV9-PHD3 group: CIH exposure was performed 3 days after TAC surgery, followed by tail vein injection of AAV9-PHD3 (5 × 10^11^ vg; GeneChem, China) 1 week later (a total of 6 weeks from the time of TAC surgery). All mice were housed in a room with an alternating light and dark cycle at 26°C and were provided free access to food and water. Animals were euthanized using cervical dislocation according to AVMA guidelines.

### Echocardiography

The mice were anesthetized with isoflurane (0.5–4%) and fixed horizontally on the operating table at 37°C. The hair on the chest of each mouse was removed with depilatory cream, and the coupling agent was applied evenly after cleaning. The MS400 probe was placed vertically on the left side of the chest of the mouse with the notch facing the head, and then the probe was rotated 45 degrees counterclockwise. Parasternal long-axis section images of the mouse heart were obtained by the small animal ultrasound imaging system (Vevo 2100, Canada). The left ventricular end-diastolic diameter (LVDd) and the left ventricular end-systolic diameter (LVDs) were measured, and the left ventricular ejection fraction (LVEF) and left ventricular fraction shortening (LVFS) were automatically calculated. Each value was measured over six consecutive cardiac cycles and averaged.

### Luciferase reporter assay

We performed a luciferase reporter gene experiment in HEK293 T cells to verify miRNA targets and conserved sites bound by miR-29c. We cloned the total length of the 3′UTR of mouse SERCA2a into the pmiR-RB-ReportTM vector to generate a SERCA2a wild-type plasmid (SERCA2a-WT) (GenePharma, China) and constructed a mutant plasmid (SERCA2a-MUT) (GenePharma, China). Vectors containing SERCA2a-WT or SERCA2a-MUT were cotransfected into HEK293T cells with miR-29c mimic or NC mimic. Vectors were transfected using Lipofectamine 3000 reagent (Invitrogen, USA). The cells were harvested 48 h after transfection, and luciferase activity was measured using a dual-luciferase assay system (Promega, USA). Regarding the relationship between SERCA2a and HIF-1α, we constructed the pGL3-SERCA2a (GenePharma, China) promoter plasmid as previously described (24). The empty cloning vector pGL3-Basic was used as a control. The vector was transfected into HL-1 cells using Lipofectamine 3000 reagent (Invitrogen, USA). Luciferase assays were performed 48 h after transfection.

### Cell culture and treatments

HL-1 cells and HEK293 T cells were kindly donated from Dr. Ma Bo. Cells were cultured in high-glucose Dulbecco's modified Eagle medium (Gibco, USA) and HL-1 cells were cultured in Claycomb medium (Sigma, USA). HL-1 cells were exposed to 0.6 μM Ang II for up to 72 h to establish a hypertrophy model concurrent with CIH exposure. Cellular CIH exposure was performed using a special intermittent cell hypoxia box (PUHE Biotechnology Company LTD, China). The cells were cultured in a humidified 5% CO^2^ incubator at 37°C. Cells were treated with CIH for 72 h (air phase set point consisting of 35 min of hypoxia followed by 25 min of reoxygenation [21% O^2^ and 5% CO^2^]). HL-1 cells were transfected with LV-PHD3 and LV-NC lentivirus vectors (GenePharma, China) following the manufacturer's instructions. The HIF-1α-PPN plasmid (GenePharma, China) was constructed according to a previously described method for cell transfection to achieve overexpression of HIF-1α under normoxia (25). For miRNA transfection, according to the manufacturer's instructions, the miR-29c inhibitor, miR-29c mimic and respective negative controls were transfected into cells with Lipofectamine 3000 reagent (Invitrogen, USA).

### Real-time PCR

mRNA expression analysis: Total RNA was isolated from mouse left ventricular tissue using the RNeasy Mini Kit (Qiagen) according to the manufacturer's instructions. cDNA was then prepared by using the Omniscript RT Kit (Qiagen, USA). qRT-PCR was performed using SYBR Green (miScript SYBR Green PCR Kit, Qiagen, USA) and the Mx3000p Real-time PCR system (Agilent). The expression of the target genes was normalized to GAPDH gene expression levels. miRNA expression analysis: miRNA was extracted from left ventricular tissue and HL-1 cells using the miRNeasy Mini Kit (Qiagen, USA) and then reverse-transcribed with the miScript II RT Kit (Qiagen, USA) using miScript HiSpec buffer. qRT-PCR was performed using SYBR Green (miScript SYBR Green PCR Kit, Qiagen, USA) and the Mx3000p Real-time PCR system (Agilent). U6 was used as the reference gene. Each sample was processed in triplicate. All data were analyzed for relative expression using the ^2−ΔΔ^Ct method. The primers used in our experiments are provided in Supplementary Table 1.

### Western blot

Protein was extracted from frozen left ventricular tissue and HL-1 cells using standard RIPA buffer. HL-1 nuclear protein extraction, using a nuclear isolation kit (P0027, Beyotime, China) according to the manufacturer's instructions, was used to determine HIF-1α protein expression. The protein concentration was determined with a BCA protein assay kit (P0012, Beyotime, China). The samples were electrophoresed on 10% SDS–PAGE gels (Gsebio) and then transferred to PVDF membranes (Immobilon). After the membranes were blocked with 5% skim milk at room temperature for 2 h, they were incubated with HIF-1α (1:1000, ab179483, Abcam, USA), sarco-/endoplasmic reticulum calcium ATPase 2a (SERCA2a) expression (SERCA2a) (1:1000, ab150435, Abcam, USA), prolyl 4-hydroxylase domain protein 3 (PHD3) (1:1000, A8001, ABclonal, China), and GAPDH (1:10000, db106, DiDiBio, China) primary antibodies overnight at 4°C. The membranes were washed three times with TBST and incubated with secondary antibody for 2 h. Finally, the blots were scanned using the LI-COR Odyssey imaging system, and ImageJ software was used for quantification.

### Histological staining

Mouse hearts were removed at the appropriate time point and then fixed with 4% polyformaldehyde. Finally, the hearts were embedded in paraffin and cut into 5 μm sections for hematoxylin and eosin (HE), wheat germ agglutinin (WGA), and Masson staining. HE staining: Sections were stained with Harris hematoxylin for 5 min and washed with 0.6% ammonia in water to return the color of the staining to blue; they were then washed again, stained with eosin for 3 min, and finally mounted and observed with a fluorescence microscope. WGA staining: The sections were stained with WGA, incubated at 37°C for 30 min in a dark incubator and observed under a fluorescence microscope. Images of the stained left ventricle and interventricular septum were obtained under a 40x microscope. The cross-sectional area of all cardiomyocytes was then quantitatively analyzed by the Image-Pro Plus 4.0 image analysis system, and the average value was obtained. Masson staining: Sections were stained with Masson's staining solution for 5 min, washed and counterstained with aniline blue solution for 5 min, mounted and observed under a fluorescence microscope. The fibrotic area was calculated by Image-Pro Plus 4.0. Phalloidin staining: Treated HL-1 cells were treated with Actin-Tracker Red-Rhodamine (C2207, Beyotime, China) following the manufacturer's instructions. The cell area measurement method was the same as above.

### Statistical analysis

Each experiment was performed at least three times with consistent results, and the data are presented as the mean ± standard deviation (SD). Differences between two groups were analyzed using a two-tailed Student's *t*-test. One-way analysis of variance (ANOVA) followed by Tukey's pairwise multiple comparisons test was used to compare multiple groups. Results with *P*-values of < 0.05 were considered statistically significant.

## Results

### CIH exposure promotes TAC-induced cardiomyocyte hypertrophy

To prolong the total duration of heart failure induced by TAC in mice, 26-G needles were used as surgical pad needles. We calculated the mortality rate of mice in the five groups. The mortality rate of mice that underwent TAC was significantly higher than that of mice that had not undergone TAC, and the difference in mortality between the three groups that underwent TAC was minimal (Figure 1B). By measuring the heart weight, body weight and tibia length of mice in each group, we obtained the mouse heart/body weight and heart weight/tibia length ratios. CIH exposure for 6 weeks alone had no effect on the mouse heart/body weight or heart weight/tibia length ratio. The heart/body weight and heart weight/tibia length ratios of the mice were increased in the TAC group. The heart/body weight and heart weight/tibia length ratios of mice were further increased in the TAC+CIH and CIH+TAC groups, but there was no difference between these two groups (Figure 1C). HE and WGA staining results showed that TAC induced hypertrophy of mouse cardiomyocytes. The cardiomyocyte hypertrophy was further aggravated in the TAC+CIH and CIH+TAC groups, with no significant difference in the level of cardiomyocyte hypertrophy between these two groups (Figure 1D). In addition, the thickness of the interventricular septal wall measured in the cross-section of the heart (Figure 1D) and the expression of cardiac pro-hypertrophy genes (Nppa and Myh7) (Figure 1E) confirmed the above results. In summary, CIH exposure before or after TAC accelerates TAC-induced cardiomyocyte hypertrophy in mice.

### CIH exposure accelerates TAC-induced left cardiac fibrosis and ventricular systolic dysfunction

**Figure 2:**
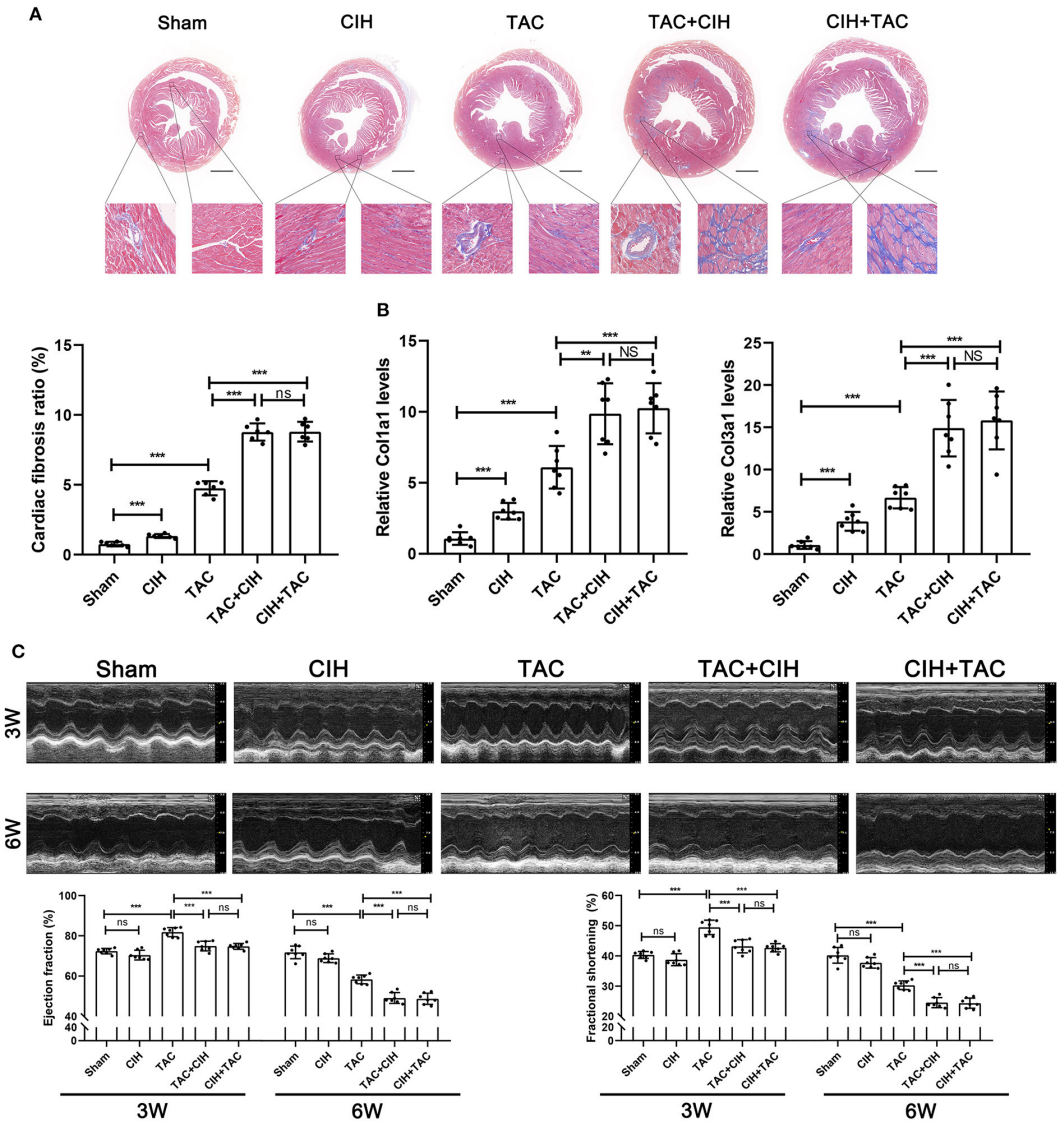
CIH exposure accelerates cardiac fibrosis and cardiac dysfunction in mice subjected to TAC. **(A)** Masson staining of heart cross-section images and representative perivascular and interstitial fibrosis images magnified in boxes; the fibrosis area ratio was quantified. *n* = 7; **p < 0.01, ***p < 0.001. **(B)** Real-time PCR analysis of relative Col1a1 and Col3a1 levels in heart tissue at week 6. *n* = 7; ***p < 0.001. **(C)** M-mode echocardiographic images of the left ventricle along the left parasternal long axis. Quantification of the LVEF and LVFS from the echocardiography data. *n* = 7; ***p < 0.001.

Cardiac fibrosis can lead to cardiac dysfunction in mice. By Masson staining, we found that the CIH group exhibited higher levels of cardiac fibrosis than the sham group (Figure 2A). Cardiac fibrosis was significantly worse in mice that underwent both CIH and TAC than in mice that underwent only TAC (Figure 2A). We determined the expression levels of cardiac fibrosis-related genes (Col1a1 and Col3a1) by qPCR, and the results were similar to those observed in the cardiac fibrosis phenotype (Figure 2B). We also performed echocardiography on mice from all groups in the third and sixth weeks. In the third week, the echocardiography data showed that the LVEF and FS in the TAC+CIH and CIH+TAC groups were lower than those in the TAC group (Figure 2C). There was no difference in echocardiography data between the CIH group and the sham group (Figure 2C). In the sixth week, a greater decrease in LVEF and FS was observed in the TAC+CIH and CIH+TAC groups than in the TAC group. There was no significant difference in mouse echocardiography data between the group exposed to CIH first and the group subjected to TAC first (Figure 2C). The LVEF and FS were slightly decreased in the CIH group, but the difference was not significant (Figure 2C). In general, CIH exposure accelerated TAC-induced left ventricular systolic dysfunction, and whether the mice were exposed to CIH first or underwent TAC first had no significant effect on the results.

### CIH exposure induces long-term elevation of HIF-1α expression levels

**Figure 3:**
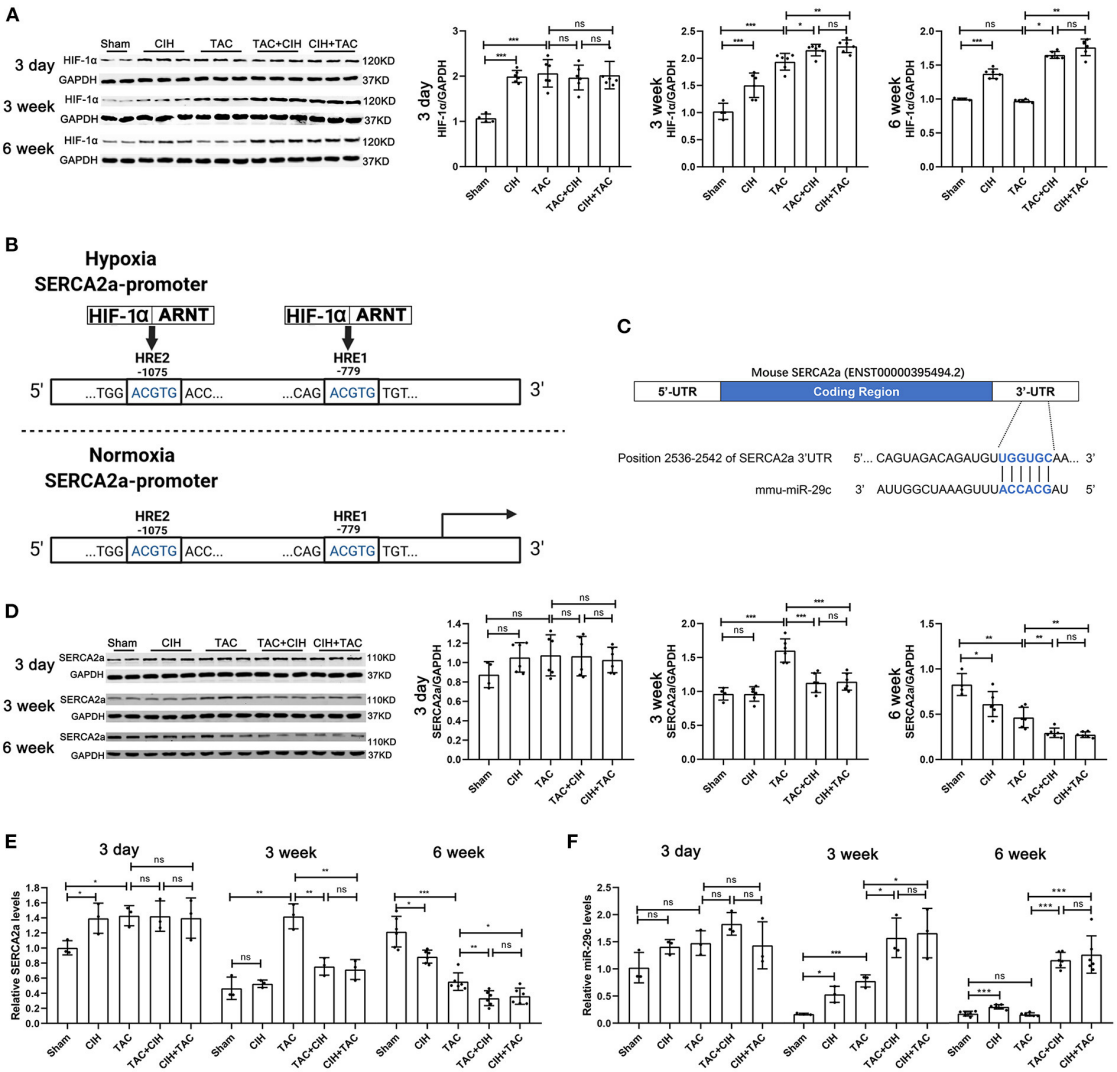
CIH exposure maintains long-term elevations in the expression of HIF-1α and induces a sustained decrease in SERCA2a levels and a sustained increase in miR-29c levels in the hearts of mice subjected to TAC. **(A)** Western blot analysis of HIF-1α expression in the heart in all groups at day 3, week 3 and week 6. *n* = 6 (sham group *n* = 4); *p < 0.05, **p < 0.01, ***p < 0.001. **(B)** Schematic diagram of the competitive binding of HIF-1 to the SERCA2a promoter region under hypoxic conditions. **(C)** Schematic representation of the miR-29c and SERCA2a mRNA binding sites. **(D)** Western blot analysis of SERCA2a expression in the heart in all groups at day 3, week 3 and week 6. *n* = 6 (sham group *n* = 4); *p < 0.05, **p < 0.01, ***p < 0.001. **(E)** Real-time PCR analysis of relative SERCA2a levels in heart tissue at day 3, week 3 and week 6. *n* = 3 (week 6 *n* = 6); *p < 0.05, **p < 0.05, ***p < 0.001. **(F)** Real-time PCR analysis of relative miR-29c levels in heart tissue at day 3, week 3 and week 6. *n* = 3 (week 6 *n* = 6); *p < 0.05, ***p < 0.001.

It was reported that in mice subjected to TAC, the expression levels of HIF-1α in the heart gradually increased within seven days after surgery, decreased after seven days, and returned to normal by 4 weeks (26). We chose to measure HIF-1α levels in the hearts of mice from all groups at 3 days, 3 weeks, and 6 weeks. The Western blot results showed that both CIH exposure and TAC increased the expression levels of HIF-1α in mouse hearts at 3 days (Figure 3A). Three weeks later, the expression of HIF-1α in the heart was higher to varying degrees in the four groups of mice subjected to different treatments than in the sham group. The TAC+CIH and CIH+TAC groups exhibited the highest expression levels of HIF-1α in the heart, but there was no significant difference in HIF-1α expression between the two groups. The TAC groups had higher levels of HIF-1α in the heart than the CIH group (Figure 3A). At 6 weeks, the expression level of HIF-1α in the heart was still higher in the CIH group than in the sham group. The level of HIF-1α in the heart was still the highest in the TAC+CIH and CIH+TAC groups, and there was no significant difference in HIF-1α expression between these two groups (Figure 3A). The HIF-1α level in the heart had returned to normal at 6 weeks in the TAC group, but it remained high in the TAC+CIH and CIH+TAC groups.

### Long-term elevations in HIF-1α expression in the heart upregulates miR-29c expression and inhibits SERCA2a expression

The expression level of SERCA2a determines the Ca^2+^ balance of cardiomyocytes and cardiac contractile function (27). During hypoxia, HIF-1 can bind to hypoxia response elements (HREs) in the SERCA2a promoter region in a competitive manner, thus inhibiting the transcription and expression of SERCA2a (Figure 3B) (24). In addition, high expression of HIF-1α has been reported to lead to the upregulation of miR-29c expression, and miR-29c was shown to negatively regulate the expression of SERCA2a (Figure 3C) (28). We verified the existence of binding sites between SERCA2a mRNA and miR-29c by bioinformatic analysis (Figure 3C). Therefore, we measured the expression levels of SERCA2a and miR-29c in the hearts of mice from all groups after 3 days, 3 weeks, and 6 weeks. After 3 days, the SERCA2a protein and mRNA levels in the hearts of mice that underwent CIH exposure or TAC were slightly increased, but not significantly (Figures 3D,E). The four groups of mice subjected to CIH exposure or TAC exhibited a small but non-significant increase in cardiac miR-29c expression (Figure 3F). After 3 weeks, there was no significant difference in SERCA2a protein or mRNA levels in the heart between the CIH group and the sham group. Interestingly, SERCA2a protein and mRNA levels were significantly increased in the TAC group, and they were significantly higher than those in the TAC+CIH and CIH+TAC groups (Figures 3E,D). This finding may have been related to adaptation to the early increase in SERCA2a expression levels in the mouse heart in response to disease (29). However, CIH exposure suppressed this adaptive change. Moreover, miR-29c levels in the hearts of mice in all groups were slightly different. The TAC+CIH and CIH+TAC groups exhibited the highest expression of miR-29c, with no difference between the groups. The expression of miR-29 in the TAC group was lower than that in the TAC+CIH and CIH+TAC groups. The expression of miR-29c was higher in the hearts of mice exposed to CIH or TAC than in the sham group (Figure 3F). After 6 weeks, the protein and mRNA levels of SERCA2a in the heart were the lowest in the TAC+CIH and CIH+TAC groups, which had similar levels. The levels of SERCA2a protein and mRNA in the heart were lower in the TAC group than in the sham group (Figures 3D,E). In contrast, the expression level of miR-29c was the highest in the TAC+CIH and CIH+TAC groups, which had similar levels. The expression level of miR-29c in the heart returned to normal in the TAC group (Figure 3F). These data combined with the results presented in section 3 of the Results indicate that, as expected, the expression of miR-29c in the heart was consistent with the expression of HIF-1α in each group. In this study, we observed that the CIH+TAC-induced long-term elevations of HIF-1α and miR-29c in the mouse heart promoted further downregulation of SERCA2a expression. In addition, the compensatory increase in SERCA2a expression in the early stage counteracted the inhibitory effect of HIF-1α and miR-29c, but under the long-term effect of chronic diseases, the body eventually decompensates, and the expression of SERCA2a is downregulated.

### HIF-1α regulates both cardiomyocyte hypertrophy and SERCA2a expression, while miR-29c only regulates SERCA2a expression

**Figure 4:**
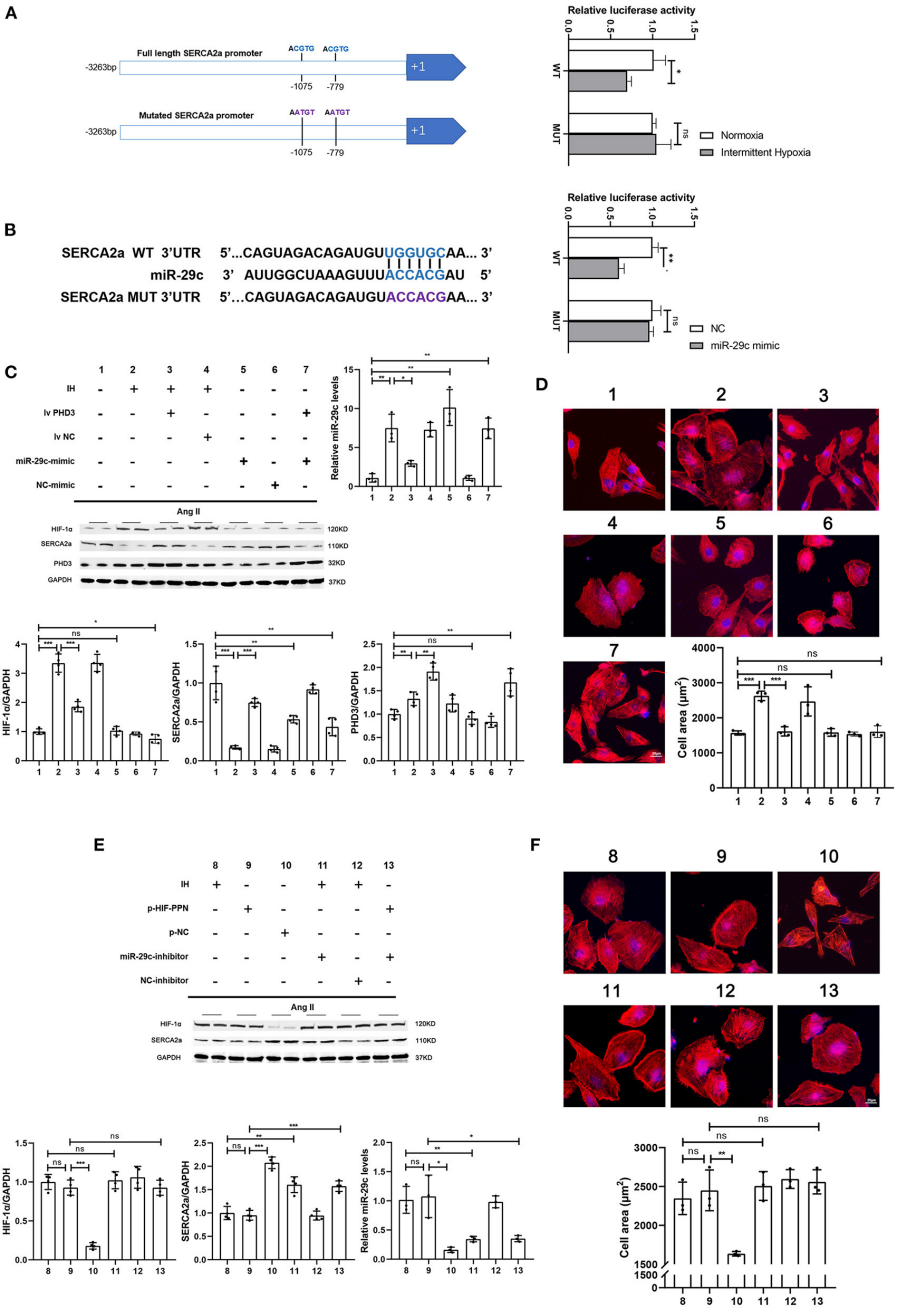
HIF-1α inhibits SERCA2a expression, promotes miR-29c expression and induces cardiomyocyte hypertrophy, while miR-29c only inhibits SERCA2a expression. **(A)** Two HIF-1 binding core sequences (HRE1 and HRE2) on the SERCA2 promoter were mutated to construct a SERCA2 promoter-luciferase reporter construct. HL-1 cells were transfected and then assayed for luciferase activity in normoxia and hypoxia. *n* = 3; *p < 0.05. **(B)** Relative luciferase activity of the SERCA2a mRNA wild-type or mutant 3'-UTR in HEK293T cells after transfection with the miR-29c mimic or NC mimic. *n* = 3; **p < 0.05. **(C)** Western blot analysis of HIF-1α, SERCA2a, PHD3 expression and real-time PCR analysis of relative miR-29c in the HL-1 cells in all groups. *n* = 4 (Western blot); *p < 0.05, **p < 0.01, ***p < 0.001; *n* = 3 (RT-PCR); *p < 0.05, **p < 0.01. **(D)** HL-1 cells treated with corresponding groups in “**(C)**” and stained with phalloidin. Then the cell area was quantified. scale bar = 20μm; ***p < 0.001. **(E)** Western blot analysis of HIF-1α, SERCA2a expression and real-time PCR analysis of relative miR-29c in the HL-1 cells in all groups. *n* = 4 (Western blot); **p < 0.01, ***p < 0.001; *n* = 3(RT-PCR); *p < 0.05, **p < 0.01. **(F)** HL-1 cells treated with corresponding groups in “**(E)**” and stained with phalloidin. Then the cell area was quantified. scale bar = 20μm; **p < 0.01.

We further verified the regulatory relationship between HIF-1α, miR-29c and SERCA2a. First, we tested the regulatory effect of HIF-1α on SERCA2a. After a previous study (24), we mutated two sites in the SERCA2a promoter that may bind to HIF-1 to construct a SERCA2 promoter-mutant luciferase reporter plasmid (Figure 4A). The SERCA2a promoter-mutant plasmid was transfected into HL-1 cells under normoxia or intermittent hypoxia, followed by a luciferase assay. The results showed that luciferase activity was not affected by intermittent hypoxia after mutation of the HIF-1-binding core sequence in the SERCA2a promoter (Figure 4A). To test the regulatory effect of miR-29c on SERCA2a, we constructed a SERCA2a 3'UTR-mutant luciferase reporter plasmid. Tool cells were cotransfected with miR-29c mimics and plasmids (Figure 4B). The results showed that after the wild-type SERCA2a 3'UTR was mutated and cotransfected with the miR-29c mimic into cells, the luciferase activity was not affected by the miR-29c mimic (Figure 4B). Then, we performed CIH exposure on the basis of an Ang II-induced cardiomyocyte hypertrophy model to establish a corresponding cellular model. Overexpression of PHD3 attenuated CIH-induced cardiomyocyte hypertrophy, with upregulation of miR-29c and downregulation of SERCA2a (Figures 4C,D). We also attempted to overexpress miR-29c under normoxia. The results showed that miR-29c only affected SERCA2a expression without causing cardiomyocyte hypertrophy regardless of whether it increased PHD3 expression (Figures 4C,D). In addition, we used the plasmid HIF-PPN (25), which encodes a HIF-1α mutant resistant to PHD degradation under normoxia. The results showed that overexpression of HIF-1α under normoxia still accelerated Ang II-induced cardiomyocyte hypertrophy, with upregulation of miR-29c and downregulation of SERCA2a (Figures 4E,F). Similar to the previous results, the miR-29c inhibitor only alleviated the downregulation of SERCA2a promoted by CIH or overexpression of HIF-1α but did not significantly alleviate cardiomyocyte hypertrophy (Figures 4E,F). These results validate the hypothesis of previous *in vivo* experiments and clarify the regulatory effects of HIF-1α and miR-29c on cardiomyocytes.

### AAV9-PHD3 can alleviate left ventricular systolic dysfunction in mice after both CIH exposure and TAC and inhibit further cardiac remodeling

**Figure 5:**
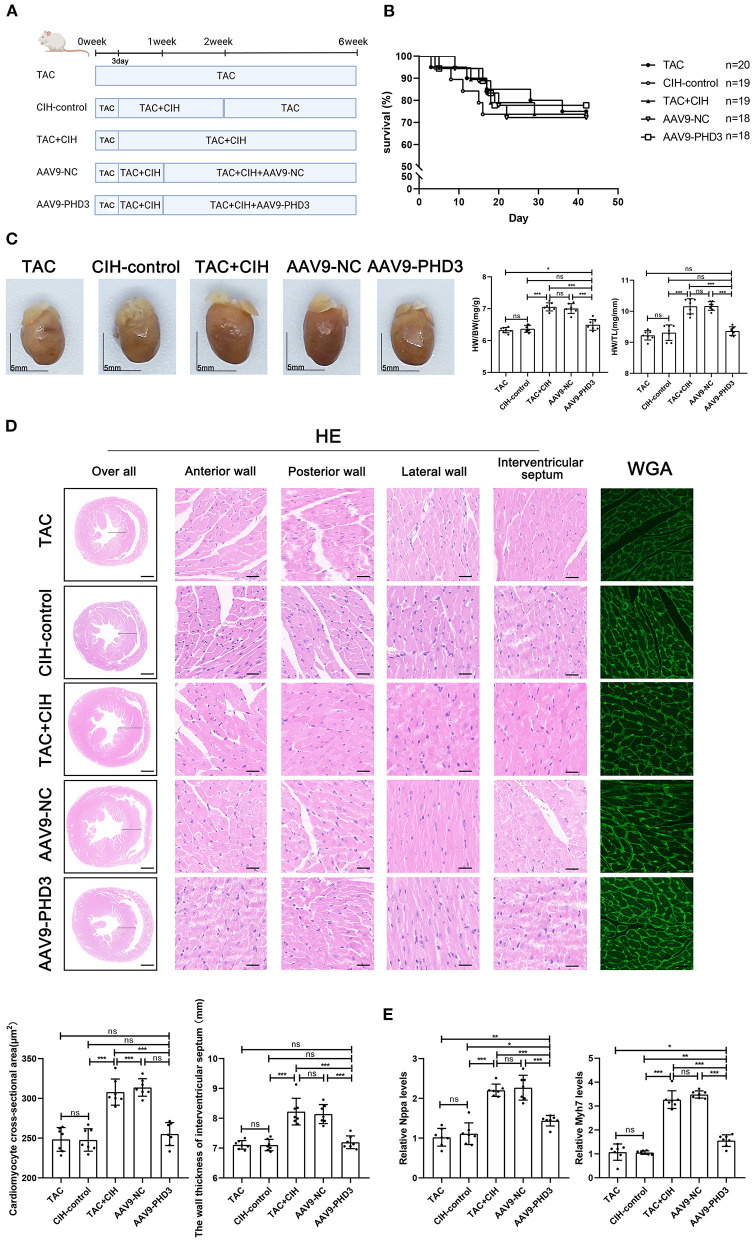
PHD3 overexpression alleviates the cardiomyocyte hypertrophy induced by CIH exposure. **(A)** Grouping and experimental protocol for each group. TAC: Transverse aortic constriction surgery was performed, followed by normoxia for 6 weeks; CIH-control: CIH exposure was performed 3 days after transverse aortic constriction surgery and CIH exposure was discontinued at the second week for a total of 6 weeks. TAC+CIH: CIH exposure was performed 3 days after transverse aortic constriction surgery for a total of 6 weeks. AAV9-NC: CIH exposure was performed 3 days after transverse aortic constriction surgery and tail vein injection of AAV9-NC on day 7 for a total of 6 weeks. AAV9-PHD3: CIH exposure was performed 3 days after transverse aortic constriction surgery and tail vein injection of AAV9-PHD3 on day 7 for a total of 6 weeks. **(B)** Survival rates of all groups; the sample size of each group is noted in the figure. **(C)** Left: representative images of hearts; right: quantification of the heart weight-to-body weight ratio and heart weight-to-tibia length ratio. *n* = 7; scale bar = 5 mm; *p < 0.05, ***p < 0.001. **(D)** HE staining and WGA staining of mouse hearts. The wall thickness of the interventricular septum was measured according to HE staining pictures. The cardiomyocyte cross-sectional area was quantified according to WGA staining. *n* = 7; scale bar = 1mm and 25μm; ***p < 0.001. **(E)** Real-time PCR analysis of relative Nppa and Myh7 levels in heart tissue at week 6. *n* = 7; *p < 0.05, **p < 0.01, ***p < 0.001.

**Figure 6:**
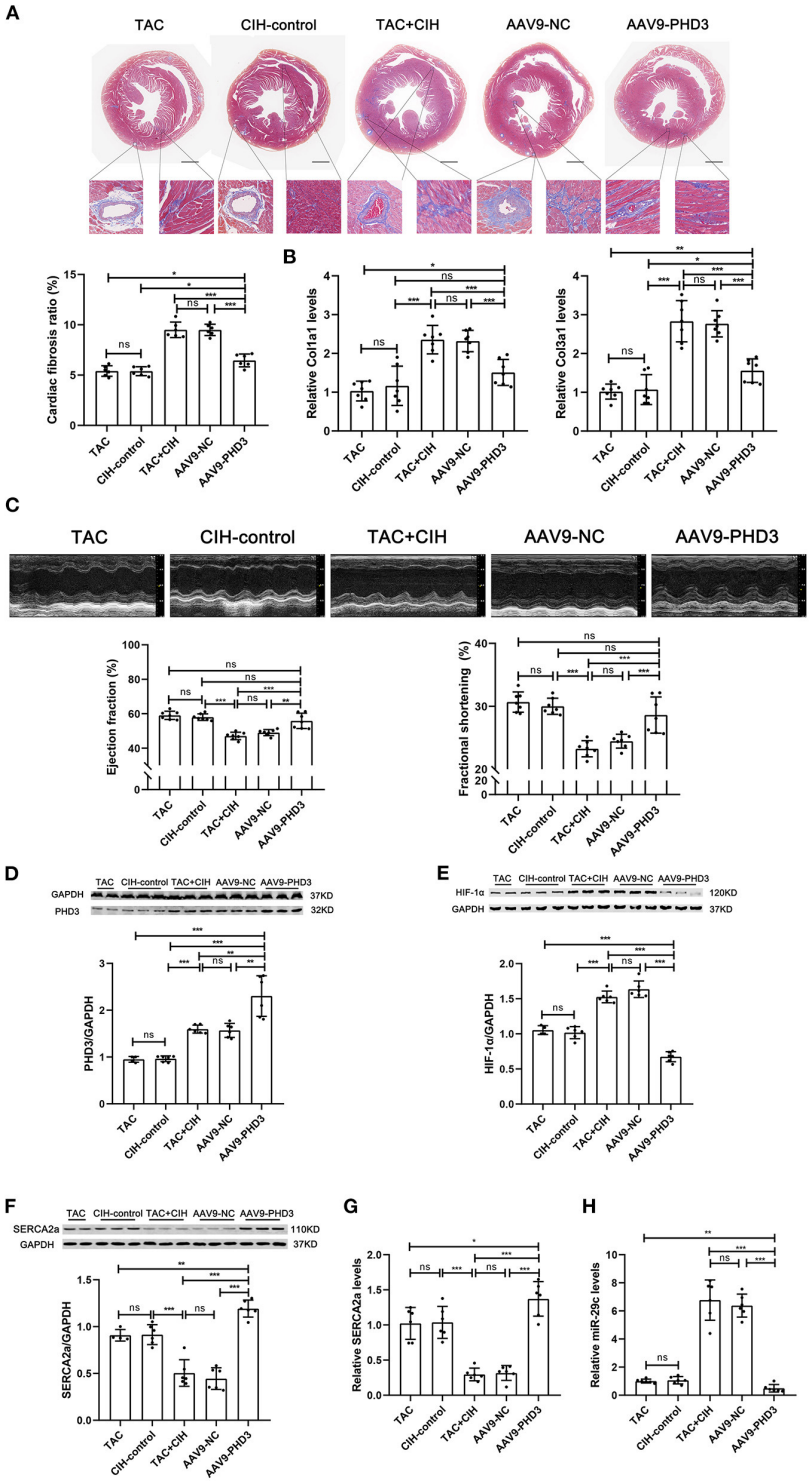
PHD3 overexpression downregulates HIF-1α and miR-29 expression to stabilize SERCA2a expression and alleviate cardiac fibrosis and cardiac dysfunction. **(A)** Masson staining of heart cross-section images and representative perivascular and interstitial fibrosis images magnified in boxes; the fibrosis area ratio was quantified. *n* = 7; ***p < 0.001. **(B)** Real-time PCR analysis of relative Col1a1 and Col3a1 levels in heart tissue at week 6. *n* = 7; ***p < 0.001. **(C)** M-mode echocardiographic images of the left ventricle along the left parasternal long axis. Quantification of the LVEF and LVFS from the echocardiography data. *n* = 7; ***p < 0.001. **(D)** Western blot analysis of PHD3 expression in the heart in all groups. *n* = 6 (TAC group *n* = 4); **p < 0.01, ***p < 0.001. **(E)** Western blot analysis of HIF-1α expression in the heart in all groups. *n* = 6 (TAC group *n* = 4); ***p < 0.001. **(F)** Western blot analysis of SERCA2a expression in the heart in all groups. *n* = 6 (TAC group *n* = 4); ***p < 0.001. **(G)** Real-time PCR analysis of relative SERCA2a levels in heart tissue. *n* = 6; *p < 0.05, ***p < 0.001. **(H)** Real-time PCR analysis of relative miR-29c levels in heart tissue. *n* = 6; **p < 0.01, ***p < 0.001.

*In vitro* experiments verified the therapeutic effect of PHD3 overexpression. Next, we used AAV9-PHD3 to increase the expression of PHD3 in the mouse heart to clarify the therapeutic effect *in vivo*. In the *in vivo* experiment presented above, our data showed that the mice that underwent both CIH exposure and TAC showed no difference in heart function after 6 weeks, regardless of whether the mice were exposed to CIH before or after TAC. Therefore, when exploring the effect of PHD3, we subjected mice to CIH exposure after TAC surgery. There was no significant difference in mortality between the five groups of mice after 6 weeks, indicating that AAV9-PHD3 had no effect on the mortality of the mice (Figure 5B). After both CIH exposure and TAC, the heart/body weight and heart weight/tibia length ratios of heart-PHD3-overexpressing mice were significantly lower, though they had no significant differences from those in the CIH control group, but the heart/body weight ratio was slightly higher than that in the TAC group (Figure 5C). HE and WGA staining results showed that further cardiomyocyte hypertrophy was inhibited in mice that were treated with AAV9-PHD3, unlike in the mice in the TAC+CIH group (Figure 5D). The thickness of the interventricular septal wall measured in the cross-section of the heart also confirmed the above result (Figure 5D). Notably, the qPCR analysis of hypertrophic genes (Nppa and Myh7) showed that the AAV9-PHD3 group still expressed slightly higher levels than the TAC and CIH control groups, but significantly lower than the TAC+CIH group (Figure 5E). Masson staining and the analysis of fibrosis-related gene expression also showed that overexpression of PHD3 in mouse heart alleviated CIH+TAC-induced cardiac fibrosis, but these mice still had more cardiac fibrosis than the TAC and CIH control groups (Figures 6A,B). Echocardiography showed that mice that underwent interrupted CIH exposure or received AAV9-PHD3 treatment exhibited higher LVEF and FS than mice in the TAC+CIH group (Figure 6C). In summary, interruption of CIH exposure and AAV9-PHD3 treatment can delay the cardiac remodeling and left ventricular systolic dysfunction induced by CIH exposure but cannot alter the cardiac remodeling and left ventricular systolic dysfunction induced by TAC.

### AAV9-PHD3 maintains the expression level of SERCA2a by inhibiting HIF-1α expression in the mouse heart

By comparing the expression levels of PHD3 in the hearts of mice injected with AAV9-vehicle and AAV9-PHD3, we verified the efficacy of AAV9-PHD3 in upregulating the expression of PHD3 in the heart (Supplementary Figure 3). We used Western blotting and qPCR to determine the levels of HIF-1α mRNA, SERCA2a mRNA, miR-29c, PHD3 protein and SERCA2a protein in the hearts of all groups of mice 6 weeks later. The results were as expected. Cardiac PHD3 was significantly elevated in the AAV9-PHD3 group (Figure 6D). Heart HIF-1α and miR-29c were the lowest in the AAV9-PHD3 group, and the levels of HIF-1α and miR-29c were similar between the CIH control and TAC groups (Figures 6E,H). In contrast, cardiac SERCA2a mRNA and protein levels were significantly higher in the AAV9-PHD3 group than in the TAC+CIH group (Figures 6F,G). These results indicate that AAV9-PHD3 increases the expression of PHD3 in the mouse heart while downregulating HIF-1α and miR-29c and preserving the expression level of SERCA2a in the heart.

## Discussion

In this study, we show that CIH exposure in combination with TAC can cause further cardiac remodeling and promote a decline in left ventricular contractile function in mice. In-depth research showed that CIH exposure in combination with TAC leads to long-term elevations in the expression of HIF-1α. HIF-1α, as a transcription factor, promotes miR-29c expression, and both HIF-1α and miR-29c can negatively regulate SERCA2a expression. Our results also showed that cardiomyocytes overexpressing PHD3 could reverse left ventricular contractile dysfunction and cardiac remodeling by inhibiting the upregulation of HIF-1α.

TAC can induce left ventricle systolic dysfunction and cardiac remodeling in mice (23). The results of previous experiments on the conditional increase/decrease in HIF-1α expression observed in mice following TAC surgery appear to be contradictory. Marion et al. subjected HIF-1α-overexpressing HIF-1α^tg^ mice to TAC for 8 weeks. Continued upregulation of HIF-1α expression accelerated cardiac dysfunction in TAC mice. In addition, they found more stable HIF-1α expression in heart samples from patients with end-stage heart failure (16). In contrast, Masanori Sano et al. conditionally knocked out Hif-1α in mouse cardiomyocytes and performed TAC surgery, and the cardiac dysfunction in Hif-1α knockout mice was more obvious 2 weeks later (30). Notably, high expression of Hif-1α promoted cardiac hypertrophy, and low expression of Hif-1α reduced the cross-sectional area of cardiomyocytes, suggesting that the pro-hypertrophic effect of HIF-1α is non-controversial. Both high HIF-1α expression at 8 weeks and low HIF-1α expression at 2 weeks after TAC surgery aggravated cardiac dysfunction, which both seem to be related to the time at which HIF-1α expression is assessed. This is similar to the role of HIF-1α in ischemic cardiomyopathy (19). Short-term HIF-1α upregulation confers some benefit, whereas long-term HIF-1α upregulation overrides the beneficial effects. Our findings emphasize the adverse effects of long-term high HIF-1α expression and do not cover any beneficial effects of short-term high HIF-1α expression.

The important role of SERCA2a in cardiac systolic function has been extensively studied. Increasing the level of cardiac SERCA2a can alleviate cardiac dysfunction (31). *In vitro*, we verified by luciferase assays that HIF-1α inhibits SERCA2a expression through both direct and indirect pathways. Cardiac injury from CIH-induced HIF-1α overexpression is only a factor that accelerates cardiac dysfunction in TAC mice. Cardiac dysfunction caused by TAC still plays an important role. TAC results in downregulation of SERCA2a expression in the heart *via* multiple pathways (32, 33). This is faster than the HIF-1α inhibition of SERCA2a. Therefore, we observed lower SERCA2a expression and worse cardiac function in the TAC group than in the CIH group at 6 weeks. Earlier in our study, we observed that HIF-1α expression was upregulated 3 days and 3 weeks after CIH exposure or TAC, at which time SERCA2a was also upregulated in the CIH and TAC groups. However, there was no simultaneous upregulation of HIF-1α and SERCA2a in our cell experiments. This reflects a strong compensatory ability in animals that can compensate for an early external pressure to increase the level of SERCA2a (34, 35). At this time, the inhibition of SERCA2a expression by HIF-1α is not enough to offset the upregulation by the body's compensation. When the external stress exceeds the body's compensatory ability, the level of SERCA2a gradually decreases, as shown by the expression of SERCA2a in the TAC+CIH and CIH+TAC groups at 3 weeks. CIH exposure in combination with TAC further upregulated HIF-1α expression, leading to cardiac decompensation. Long-term elevations in the expression of HIF-1α led to a more obvious impairment in cardiac function at 6 weeks. In addition, we observed that mice subjected to CIH alone for 6 weeks showed an increase in the levels of the hypertrophic factor Myh7 and cardiac fibrosis, although there was no significant decrease in left ventricular function. This finding suggests that 6 weeks of elevated HIF-1α expression in normal mice also induced some cardiac damage, but within the compensable range of the heart. In summary, our study once again demonstrates that long-term activation of HIF-1α accelerates the development of cardiac injury in mice.

Under normoxic conditions, PHD3 hydroxylates 2 specific proline residues in the oxygen-dependent degradation (ODD) domain of HIF-1α, and then von Hippel–Lindau protein mediates HIF-1α degradation. Under hypoxic conditions, PHD activity is inhibited, and HIF-1α escapes hydroxylation and is transported into the nucleus to play a regulatory role in transcription (36). Cardiac PHD3 activation mimics the inhibition of HIF-1α activation under normoxic conditions, which is more similar to what is observed under physiological conditions. In this experiment, we first overexpressed PHD3 in a model of cardiomyocyte injury induced by the combination of Ang II and CIH to verify the therapeutic effect. Then we verified the therapeutic effect of AAV9-PHD3 on cardiac systolic dysfunction and cardiac remodeling induced by CIH exposure in mice with overload stress. We also observed that CIH accelerated cardiac fibrosis in TAC mice. We alleviated the cardiac fibrosis in TAC mice by overexpressing PHD3 in the heart, including but not limited to cardiomyocytes. Similarly, in our previous study (21), CIH activated HIF-1α, which increased endothelial mesenchymalization, leading to cardiac fibrosis. Notably, miR-29c is also one of the influencing factors of cardiac fibrosis (37). However, this study mainly focused on cardiomyocytes and did not further elaborate on the specific reasons. We hope to explore these issues in more depth in follow-up studies.

Although this seems to be a satisfactory result, we noticed that AAV9-PHD3 treatment reduced the adverse cardiac effects of CIH exposure but did not have a therapeutic effect on the TAC-induced basic heart damage. We also observed that PHD3 overexpression did not reduce mortality in mice. This may be due to the limited effect on mortality in mice with an EF of approximately 50%, and more deaths were due to infection or complications from TAC surgery. Between the TAC+CIH, CIH+TAC, and TAC groups, there were differences in cardiac function but no significant difference in mortality. However, we think that the effect of PHD3 on mortality may become visible in the animal model after longer experimental times. In addition, there are some limitations to our study. First, because of the cardiovascular protective effect of estrogen in adult female mice, we only selected male C57BL/6 mice, which are more sensitive to CIH exposure. Second, the role of SECRA2a and PHD3 is affected not only by their expression levels but also by SECRA2a and PHD3 activity. Finally, due to the limitations of experimental conditions, we could not directly verify the contractile function of cells *in vitro* but chose to use SERCA2a expression for verification.

## Conclusion

**Figure 7:**
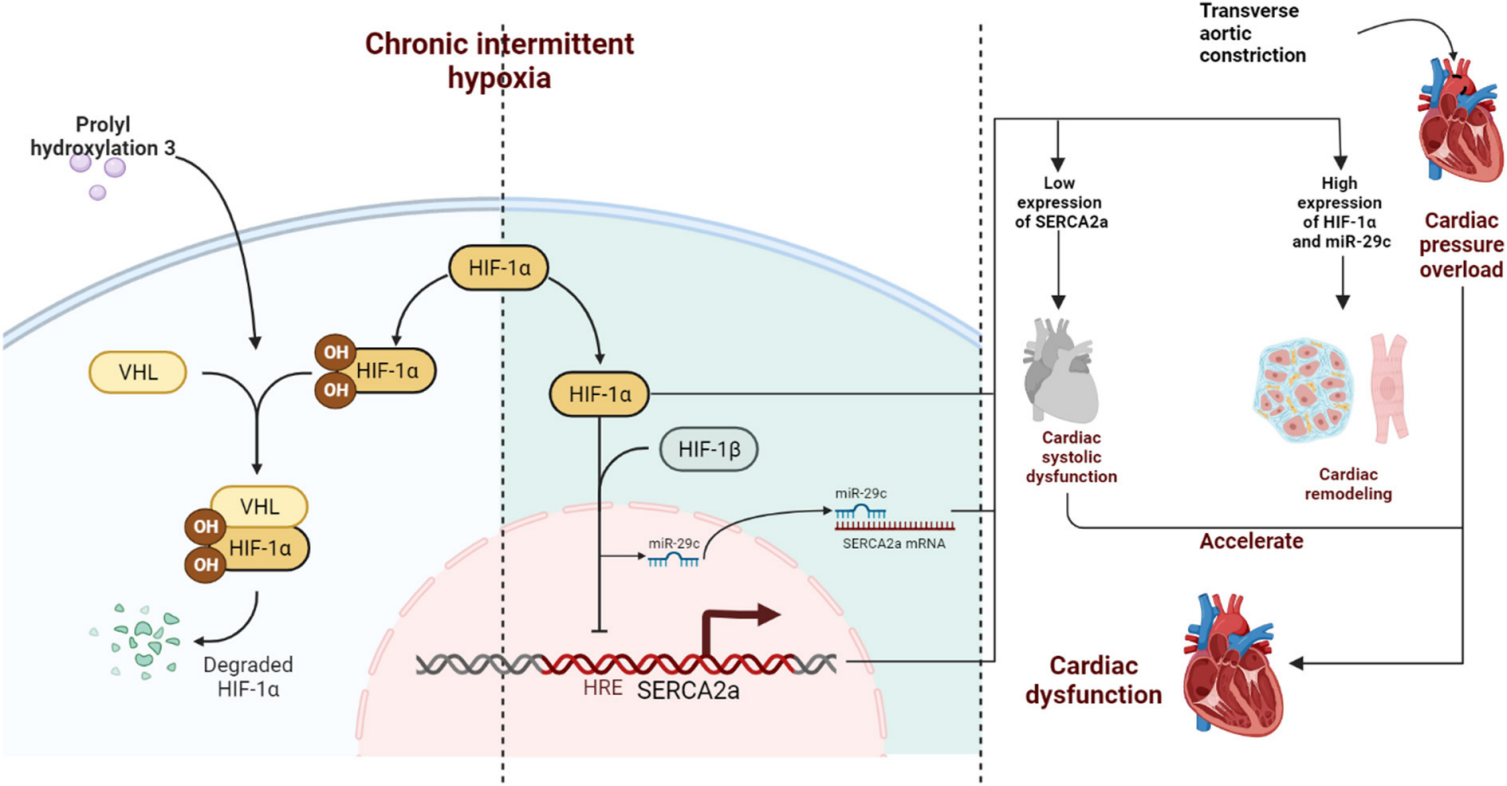
Schematic diagram. CIH exposure promotes long-term abnormalities in the expression of HIF-1α in mice subjected to TAC, further inducing continuous upregulation of miR-29c expression. Together, HIF-1α and miR-29c inhibit the expression of SERCA2a in the nucleus and cytoplasm in cardiomyocytes accelerating left ventricular systolic dysfunction. In addition, HIF-1α promotes cardiac remodeling, which ultimately synergistically accelerates cardiac dysfunction. PHD3 overexpression in the heart during CIH can lead to degradation of HIF-1α in a timely manner and reduce cardiac damage caused by CIH exposure.

Our findings, which are shown in the schematic illustration (Figure 7), identify therapeutic targets for CIH exposure-induced upregulation of HIF-1α expression, which accelerates TAC-induced left ventricular systolic dysfunction and cardiac remodeling. We also demonstrate that high expression of myocardial PHD3 promotes the downregulation of HIF-1α induced by intermittent hypoxia and that downregulation of HIF-1α expression leads to transcriptional repression of miR-29c, upregulating SERCA2a expression and ultimately alleviating cardiac remodeling and dysfunction.

## Data availability statement

The original contributions presented in the study are included in the article/Supplementary material, further inquiries can be directed to the corresponding author/s.

## Ethics statement

The animal study was reviewed and approved by Animal Care and Use Committee of Southeast University School of Medicine.

## Author contributions

J-YT and XX: conception and design of the research and drafting manuscript. XX, P-HZ, F-CY, TW, S-NL, and QW: acquisition of data and analysis and interpretation of data. XX and P-HZ: statistical analysis. J-YT: obtaining funding. All authors read and approved the final manuscript.

## Funding

This study was supported by Natural Science Foundation of Jiangsu Province (BK20210231).

## Conflict of interest

The authors declare that the research was conducted in the absence of any commercial or financial relationships that could be construed as a potential conflict of interest.

## Publisher's note

All claims expressed in this article are solely those of the authors and do not necessarily represent those of their affiliated organizations, or those of the publisher, the editors and the reviewers. Any product that may be evaluated in this article, or claim that may be made by its manufacturer, is not guaranteed or endorsed by the publisher.

## Supplementary material

Supplementary Figure 1 Flow chart of the experimental process. Surgical death: Mice that died during TAC surgery due to vascular rupture or pneumothorax and other causes. Surgical failed: TAC surgical ligation was wrongly positioned or the operation time was too long, the mice suffered from respiratory disturbance and were forced to stop the operation.

Supplementary Figure 2 Flow chart of the experimental process.

Supplementary Figure 3 Left: CIH exposure after TAC surgery, tail vein injection of AAV9-PHD3 on week 1, and PHD3 protein expression was measured in cardiac tissue on week 6. Right: PHD3 protein expression in heart tissue after 4 weeks of AAV9-PHD3 tail vein injection under normoxia.

Supplementary Table 1 Primer sequences used for qRT-PCR.
