# Supplementary figures and images for: Chronic intermittent hypoxia accelerates cardiac dysfunction and cardiac remodeling during cardiac pressure overload in mice and can be alleviated by PHD3 overexpression
Source: Front Cardiovasc Med. 2022 Sep 12;9:974345. doi: 10.3389/fcvm.2022.974345 (PMC9510693; doi:10.3389/fcvm.2022.974345)

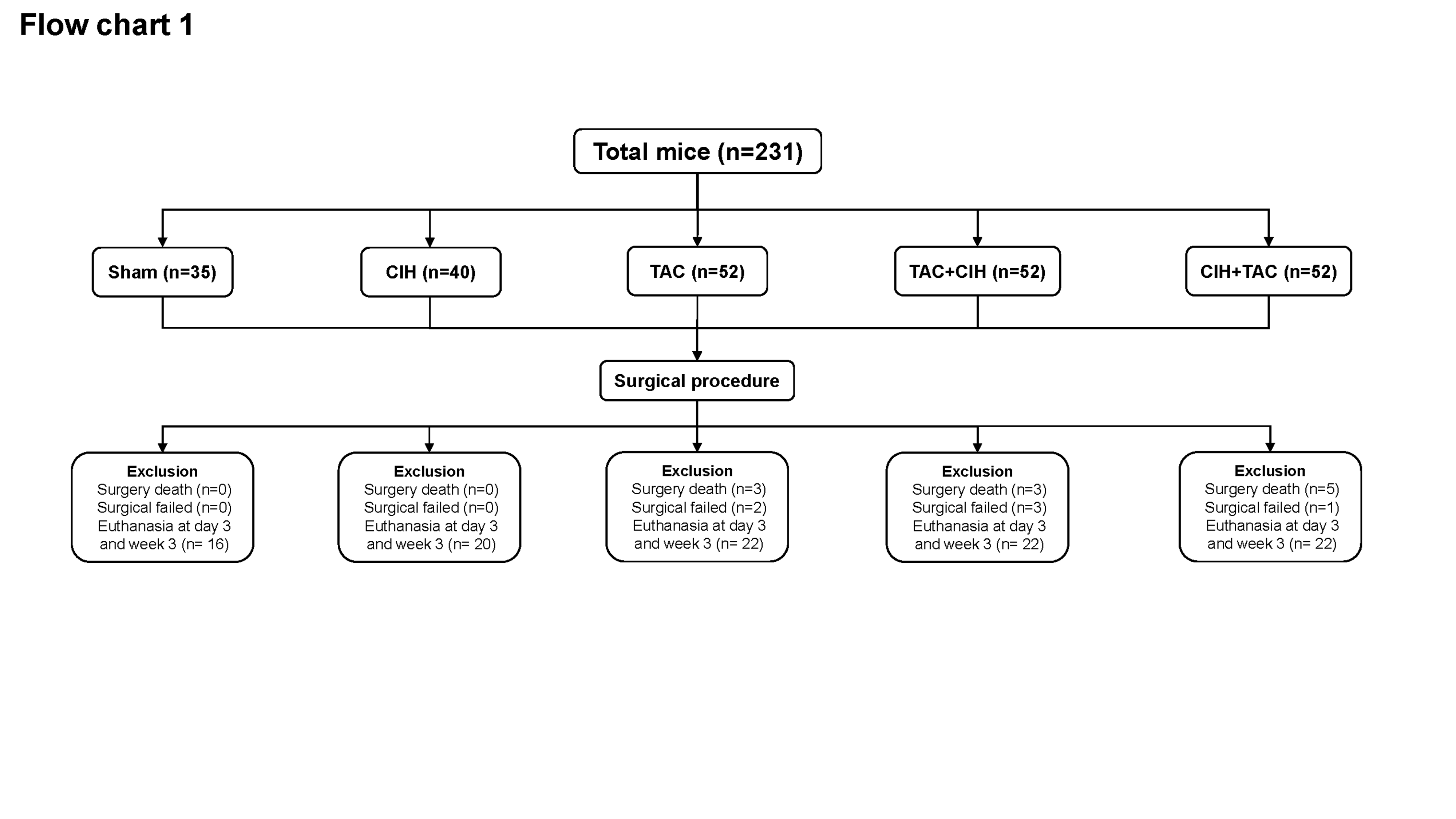

Supplement: Supplementary Figure 1 — Flow chart of the experimental process. Surgical death: Mice that died during TAC surgery due to vascular rupture or pneumothorax and other causes. Surgical failed: TAC surgical ligation was wrongly positioned or the operation time was too long, the mice suffered from respiratory disturbance and were forced to stop the operation. [file Image_1.TIF]

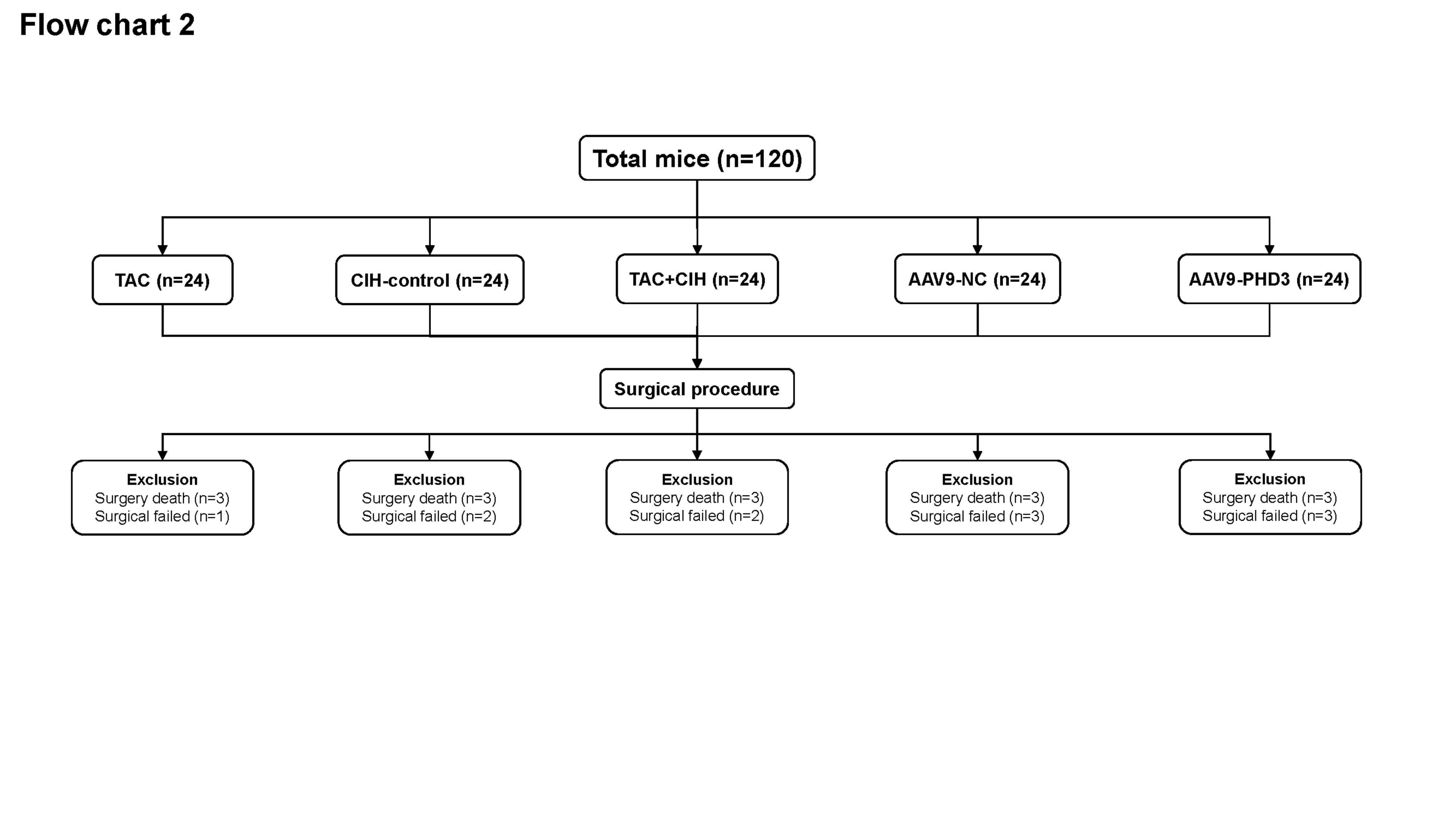

Supplement: Supplementary Figure 2 — Flow chart of the experimental process. [file Image_2.TIF]

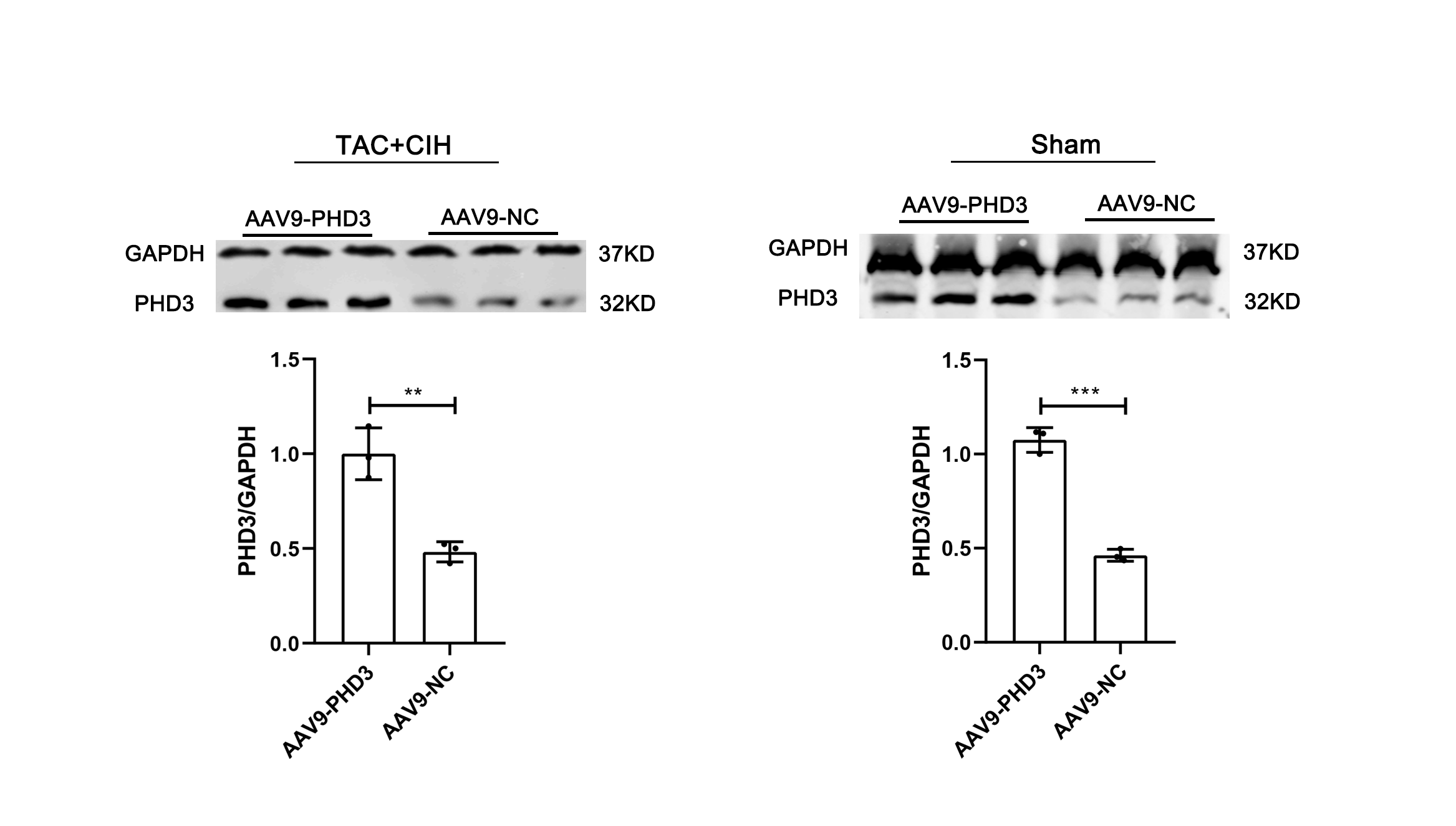

Supplement: Supplementary Figure 3 — Left: CIH exposure after TAC surgery, tail vein injection of AAV9-PHD3 on week 1, and PHD3 protein expression was measured in cardiac tissue on week 6. Right: PHD3 protein expression in heart tissue after 4 weeks of AAV9-PHD3 tail vein injection under normoxia. [file Image_3.TIF]
