# Supplementary material for: Chronic intermittent hypoxia accelerates cardiac dysfunction and cardiac remodeling during cardiac pressure overload in mice and can be alleviated by PHD3 overexpression
Source: Front Cardiovasc Med. 2022 Sep 12;9:974345. doi: 10.3389/fcvm.2022.974345 (PMC9510693; doi:10.3389/fcvm.2022.974345)
Supplement: Supplementary Table 1 — Primer sequences used for qRT-PCR. [file Table_1.DOCX]

**Table S1. Primer sequences used for qRT-PCR.**

| Primer Set | Primers |
| --- | --- |
| GAPDH | F: AGGTCGGTGTGAACGGATTTG  R: TGTAGACCATGTAGTTGAGGTCA |
| Nppa | F: GCTTCCAGGCCATATTGGAG  R: GGGGGCATGACCTCATCTT |
| Myh7 | F: ACTGTCAACACTAAGAGGGTCA  R: TTGGATGATTTGATCTTCCAGGG |
| Col1a1 | F: GCTCCTCTTAGGGGCCACT  R: CCACGTCTCACCATTGGGG |
| Col3a1 | F: CTGTAACATGGAAACTGGGGAAA  R: CCATAGCTGAACTGAAAACCACC |
| SERCA2a | F: TAAATGCCCGCTGTTTTGCT  R: TTGTCATCTGCCAGGACCAT |
| U6 | F: GTGCTCGCTTCGGCAGCACATAT  R: AAAATATGGAACGCTTCACGAA |
| miR-29c-3p | F: AACACGTGTAGCACCATTTGAA  R: CAGTGCAGGGTCCGAGGT |
